# An analysis of the poverty reduction effects on land transfer: Evidence from rural areas in China

**DOI:** 10.1371/journal.pone.0298243

**Published:** 2024-02-20

**Authors:** Wenguang Yu, Guofeng Guan, Yifan Wang, Qi Wang

**Affiliations:** 1 School of Insurance, Shandong University of Finance and Economics, Jinan, Shandong, China; 2 School of Statistics and Mathematic, Shandong University of Finance and Economics, Jinan, Shandong, China; 3 Department of Agricultural and Applied Economics, College of Agriculture and Life Sciences, Virginia Polytechnic Institute and State University, Blacksburg, VA, United States of America; Loughborough University, UNITED KINGDOM

## Abstract

This paper develops a multidimensional poverty index (MPI) evaluation system using multiple measures. We use the China Family Panel Study (CFPS) data to build balanced panel data from 2012 to 2018. Employing the probit model to analyze the impact of land transfer on relative poverty incidence, as well as utilizing the two-way fixed effects model and the logit model, we approach the issue from the perspective of multidimensional relative poverty identification. Our study indicates a decrease in relative poverty among rural households since 2012. Nonetheless, the overall incidence of relative poverty among rural households in China remains high at 20.6%, highlighting the severity of this issue in rural China. Moreover, we examine the heterogeneity of the poverty reduction effects of land transfer-in and land transfer-out. Land transfer can significantly reduce the incidence of relative poverty among rural households, with distinct mechanisms for land transfer-in and land transfer-out. Land transfer-in primarily reduces the relative poverty incidence of rural households through the education, housing, and land dimensions, while land transfer-out focuses on the quality-of-life dimension. Overall, land transfer-out has a more significant poverty reduction effect than land transfer-in. Furthermore, our study reveals that the reduction effect of land transfer on the incidence of relative poverty among rural households persists for at least two years, but by the fourth year, this effect disappears.

## 1. Background

As a long-standing societal concern, poverty remains a pivotal topic in development economics. In the pursuit of eradicating poverty and fostering prosperity within society, China embarked an eight-year battle against poverty, culminating in a remarkable victory. In 2021, 99 million people were lifted out of poverty under the current poverty standard, marking the successful elimination of absolute poverty. Subsequently, China’s poverty alleviation strategy transitioned from solving absolute poverty to relative poverty. The Fourth Plenary Session of the 19th Central Committee (2019) proposed resolutely winning the battle against poverty and establishing a long-term mechanism to address relative poverty. Identifying and measuring relative poverty holds significant academic and practical value for tackling this issue.

Different definitions of relative poverty have been proposed by leading economists, and their contributions have significantly shaped the development of this field. Townsend (1962) was among the first to suggest that poverty is not solely the absence of necessities but also the lack of resources necessary for individuals, families, and social organizations to secure food, shelter, recreation, and participation in social activities [1]. This insufficiency prevents them from reaching the average standard of living prescribed by societal norms, resulting in their exclusion from common ways of life and social activities. Sen (1976) expanded on this concept by defining poverty as a deprivation of a person’s "viability", which includes a range of functions, such as freedom from hunger, freedom from disease, and access to education [2]. This approach to defining poverty is commonly referred to as the capability approach. In the discourse on relative poverty theory led by scholars like Townsend and Sen, the methods for measuring relative poverty have evolved. The current academic community broadly embraces two main approaches in defining relative poverty: (i) setting a certain percentage of the mean or median income as the relative poverty line and determining relative poverty based on whether an individual’s income falls below this line; (ii) constructing comprehensive multidimensional indicators to measure relative poverty.

Some scholars use the first method to calculate the relative poverty line, which may have been influenced by the EU’s introduction of setting it at 60% of the median disposable income per capita. If the household income falls below this percentage, it is in a state of in relative poverty. However, scholars who use such methods to define relative poverty disagree on whether to use the mean or median income. Fuchs (1967) argued that the measurement of poverty should be based on a comparison of required resources, and an individual or household is in poverty if its resources do not reach the poverty line based on the reference group [3]. He was the first to propose using 50% of median income as the relative poverty line. In comparison, Drewnowski (1977) suggested using 50% of the mean income as the relative poverty criterion [4]. Since then, more studies have also employed 50% or 60% of the median income level to define relative poverty, as seen in Thompson (2013), Gustafsson and Sai (2020), Weon and Hye-ryun (2020), Zou et al (2023) [5–8].

Since Sen (1976) introduced the capability approach to poverty, research on relative poverty has gradually shifted from a single dimension of income to multiple dimensions [2]. However, how to capture and measure this multidimensional capability poverty has become the focus of scholars’ attention. Alkire and Foster (2011) proposed a novel approach to measuring relative poverty from a multidimensional perspective, known as the A-F ‘dual cutoff’ identification approach (the AF method) [9]. The AF method stands as a landmark in measuring relative poverty from a multidimensional perspective, emphasizing multidimensional and comprehensive identification and measurement of relative poverty rather than relying solely on the income criterion. Building upon the AF method, Alkire and Santos developed the multidimensional poverty index (MPI), which comprises ten indicators spanning three dimensions: health, education, and living standards. The MPI gained recognition from the United Nations Development Programme (UNDP) and has been widely used in the global practice of multidimensional poverty measurement (OPHI, 2022). Scholars have adopted the MPI to some extent, depending on the data availability and their research focus. Notably, the existing literature has incorporated additional dimensions such as employment, healthcare, and land into the MPI to offer a more comprehensive measure of relative poverty, Dickerson and Popli (2018), Fransman and Yu (2019), Alkire and Fang (2019), Aguilar and Sumner (2020), Zhang et al (2021) [10–14].

## 2. Introduction

Currently, resources are unevenly distributed under the urban-rural dual structure in China, and relative poverty is significantly higher in rural China than in urban areas, Alkire and Fang (2019), Deng and Zheng (2022), Wei and Tang (2022) [12, 15, 16]. Therefore, addressing relative poverty in rural areas should be the primary focus of China’s ongoing poverty alleviation efforts. Land, as an essential means of production and livelihood security for farmers, has a significant impact on the rural economy’s development. With the reform of China’s property rights system and land management rights, land transfer, an important aspect of this reform, has garnered attention from scholars in China and abroad due to its influence on the rural economy, particularly in addressing farmers’ poverty. China’s rural land operates under a system of collective ownership and farmer-managed contracts. In this context, land transfer, as discussed in this paper, does not involve the transfer of ownership rights but rather the transfer of land management rights to other parties with the farmers’ voluntary consent. Land transfer-out involves renting one’s own land for cultivation, receiving rent, and allowing farmers to work elsewhere. Conversely, land transfer-in entails renting land from others for cultivation, paying rent to the landowners, and increasing income by cultivating additional land.

Previous studies have also incorporated the land dimension when using the MPI method to measure relative poverty and explored the impact of land transfer on absolute poverty reduction, Keswell and Carter (2014) [17] and the alleviation effect on relative poverty, Liu and Wang (2020) [18]. They found that land transfer-in and transfer-out can effectively alleviate poverty, Zhou et al (2020), Zuo and Lu (2020), Li et al (2021) [19–21]. However, these studies have several limitations: (i) some of the literature continue to rely on the relative poverty line method, which does not comprehensively measure relative poverty from a multidimensional perspective; (ii) there exists a degree of endogeneity of land transfer and a potential bidirectional causality between land transfer and household relative poverty; (iii) while some studies have concluded that land transfer can alleviate relative poverty, they have mainly relied on cross-sectional data, which restricts the analysis of the long-term effects of land transfer on the relative poverty of rural households. To address these limiations, we propose adopting the instrumental variables approach to overcome the endogeneity problem between land transfer behavior and household relative poverty. We use panel data to evaluate the long-term impact of land transfer on the incidence of relative poverty among rural households and explore in-depth the mechanism through which land transfer alleviates relative poverty.

The rest of the paper is structured as follows. Section 3 describes the methodology and the data. Section 4 presents the model. Section 5 evaluates the poverty reduction effect and performs sensitivity tests. Section 6 performs further analysis. Finally, Section 7 presents the conclusions.

## 3. Methodology

### 3.1 Relative poverty identification method

Assuming that the total number of rural households is *N*, (a constant in period *T*), there are *d* dimensions measuring the MPI. Then *x*^*t*^_*ij*_ denotes the value taken by the *i* th household in period *t* in *j* th dimension, i.e:

$${x^{t}}_{ij}\geq 0,\;(i=1,2,\ldots ,N;j=1,2,\ldots ,d;t=1,2,\ldots ,T)$$ (1)

For each dimension *j*, we use vector *z*_*j*_ = (*z*_1_,…,*z*_*d*_) to denote the deprivation cutoff or minimum achievement level. To ensure comparability of poverty measures across different periods, we assume that the deprivation cutoff for each indicator remains constant for all *T* periods.

Assume that $g_{ij}^{t}(z)$ is a unidimensional identification function. When *x*^*t*^_*ij*_<*z*_*j*_, *g*^*t*^_*ij*_(*z*) = 1, it indicates that the *i*th household is deprived in *j*th dimension. Otherwise, $g_{ij}^{t}(z)=0$, signifying that the household is not deprived in that dimension. We set *k*(0<*k*≤1) as the poverty cutoff of the MPI. Additionally, let *w*_*j*_(0<*w*_*j*_≤1) represents the weights that indicate relative importance of deprivation in each dimension with $\sum_{j=1}^{d}w_{j}=1$. The variable $c_{i}^{t}$ denotes the total deprivation score of the *i* th household in each dimension at time *t*, such that ${c^{t}}_{i}=\sum_{j=1}^{d}w_{j}{g^{t}}_{ij}(z)$, with a larger $c_{i}^{t}$ indicating higher deprivation. Finally, let $I_{i}^{t}$ be the identification function of multidimensional poverty at time *t*. If $c_{i}^{t}\geq k$, we have $I_{i}^{t}=1$, and the *i* th household is identified as multidimensionally poor. Otherwise, $I_{i}^{t}=0$ and the household *i* is not identified as multidimensionally poor.

### 3.2 Dimensions, indicators, weights, and cutoffs

Building upon the capability approach to poverty proposed by Sen, this paper refines and enhances the internationally recognized MPI by considering domestic research results and data availability. It establishes eight dimensions and sixteen indicators covering education, health, employment, healthcare, quality-of-life, housing, land, and income (Table 1).

**Table 1:** Multidimensional poverty dimensions, indicators, weights and cutoffs.

Dimensions	Indicators	Indicator descriptions	Weights	Cutoffs
Education (1/8)	Years of schooling	Average years of schooling of household members aged > 16	1/16	9 years
	School attendance	Children (6–16 years old) go to school (yes = 1, no = 0)	1/16	1
Health (1/8)	BMI	Proportion of adults in the households with BMI between 18.5 and 28	1/24	50%
	Hospitalization	No household member has been hospitalized in the past year (yes = 1, no = 0)	1/24	1
	Self-rated health status	None of the household members self-rated their health as very unhealthy (yes = 1, no = 0)	1/24	1
Employment (1/8)	Employment status	Among household labor force (16–60 years old) at least 1 person works (yes = 1, no = 0)	1/16	1
	Formal employment	Household members (16–60 years old) have formal employment (yes = 1, no = 0)	1/16	1
Health care (1/8)	Medical insurance	Proportion of family members enrolled in medical insurance	1/8	100%
Quality-of-life (1/8)	Water	Water: Water used at home is tap water, barreled water, distilled or filtered water (yes = 1, no = 0)	1/32	1
	Electricity	Electricity access in the home (yes = 1, no = 0)	1/32	1
	Cooking fuel	Household uses clean fuel for cooking (yes = 1, no = 0)	1/32	1
	Value of durable goods	Total value of durable goods	1/32	1000 RMB
Housing (1/8)	Owned housing	Household owns housing or can obtain housing from government or employment (yes = 1, no = 0)	1/16	1
	Living space	Household living space per capita	1/16	12 m^2^
Land (1/8)	Land status	The household can obtain one of arable land, forest land, pastureland, or water pond from collective allocation (yes = 1, no = 0)	1/8	1
Income (1/8)	Net income per capita	Net income per capita of households (smoothed to 2011 by the index of consumption level of rural residents)	1/8	2300 RMB

Note: BMI = weight (Kg)÷ height (m)^2; Formal employment is defined as the population employed in government agencies, state-owned institutions and enterprises, triple-funded enterprises and larger private enterprises, and other large units. The population employed in private and individual enterprises (except for enterprises above a certain size) and other small units is classified as informal employment.

Following the practice of the UNDP and OPHI, this paper assigns equal weight to all dimensions when calculating the MPI index. The deprivation cutoff for the MPI is set at 0.33, designating households with an MPI exceeding 0.33 as relatively poor households.

### 3.3 Data selection

The empirical data for this study is sourced from the China Family Panel Studies (CFPS), a comprehensive database that tracks individual, household, and community-level data. Moreover, it focuses on various aspects of Chinese residents’ economic and non-economic well-being, including economic activity, educational outcomes, family relationships, family dynamics, population migration, health, and other research topics. The survey sample spans 25 provinces (municipalities and autonomous regions) and includes all household members in the sample households. The database contains many indicators that reflect the multidimensional poverty of rural households, such as education, employment, and quality-of-life. For this paper, we select samples in 2012, 2014, 2016, and 2018 to investigate the impact of the land transfer on the relative poverty of rural households in China. This data can be downloaded for free on http://www.isss.pku.edu.cn/cfps/.

To obtain multi-level data for our research, we process the data as follows: we exclude samples that consist solely of urban household members, retaining only agricultural households. Then, we exclude samples that enter the dataset after 2012 and those that exit between 2012 and 2018. Finally, we retain balanced panel data with a valid sample of 6867 households per year.

### 3.4 Identification results of relative poverty

**Fig 1:**
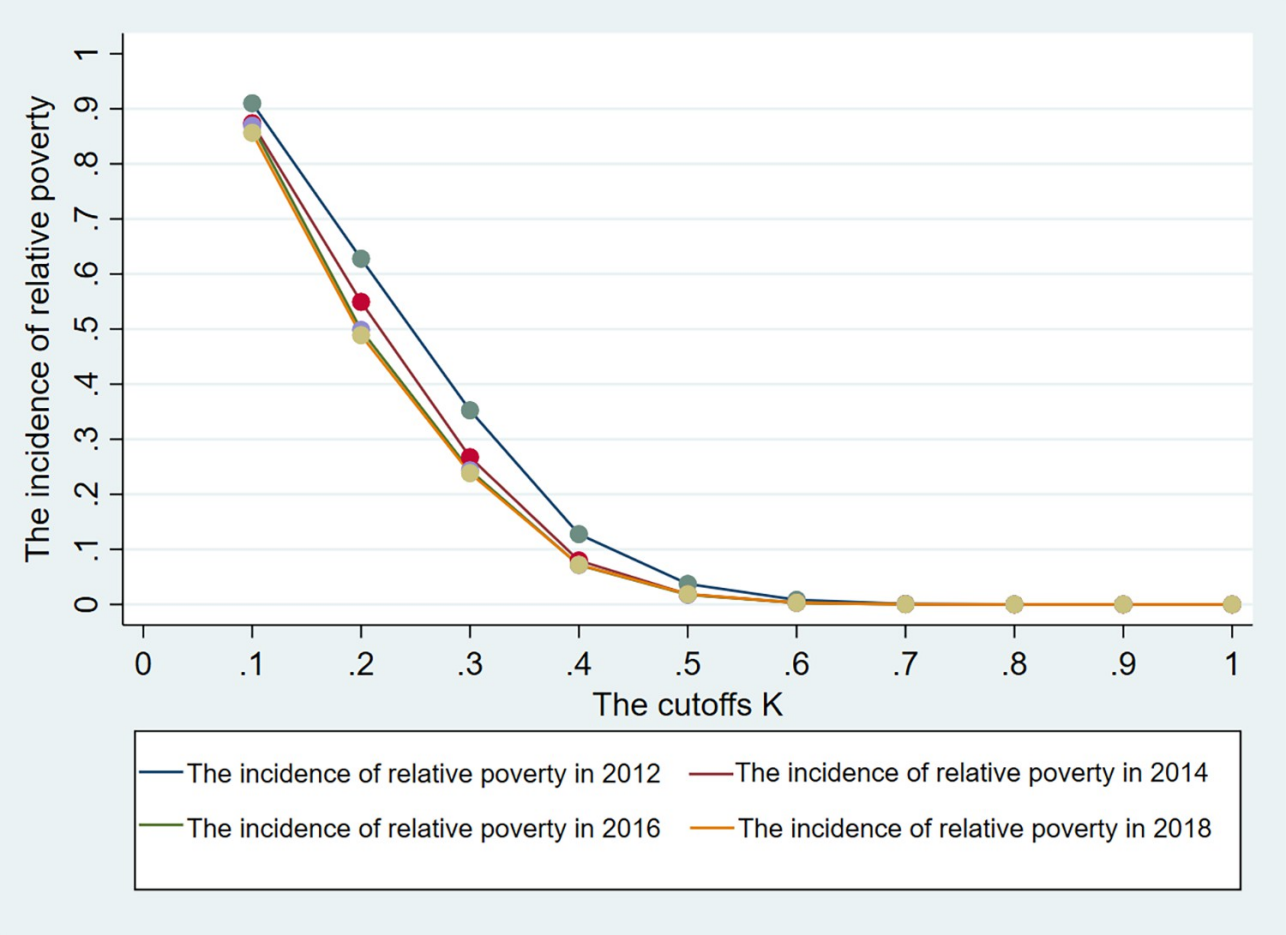
Changes in relative poverty incidence under different cutoffs K.

According to the MPI calculation method, rural households are identified as relatively poor using different cutoffs. The four curves in Fig 1 illustrate the relative poverty incidence in various years. Fig 1 reveals two key observations: (i) as the relative poverty cutoff increases, the proportion of households identified as relatively poor deceases and (ii) under the same relative poverty cutoff, the incidence of relative poverty steadily declines year after year, yielding successful outcomes of poverty eradication efforts.

## 4. Empirical model setting

### 4.1 Setting of the econometric model

This paper constructs the following two-way fixed effects model and probit model based on the least squares method.

$$Poor_{it}=\alpha_{0}+\beta transfer_{it}+\sum_{n}\alpha_{m}{X^{m}}_{it}+\phi_{year}+\eta_{i}+\delta_{it},\;i=1,2,\ldots ,N$$ (2)

$$\mathrm{Pr}obit(Poor_{it}=1)=\alpha_{0}+\beta transfer_{it}+\sum_{n}\alpha_{m}{X^{m}}_{it}+\phi_{year}+\theta_{province}+\delta_{it},\;i=1,2,\ldots ,N$$ (3)

*Poor*_*it*_ denotes whether the *i*th rural household is relatively poor in period *t*, with 1 denoting relative poverty and 0 indicating otherwise. *transfer*_*it*_ represents whether the *i*th rural household is involved in land transfer during period *t*, with 1 representing participation and 0 indicating non-participation. *β* is the regression coefficient of *transfer*_*it*_. *X*^*m*^ comprises control variables affecting the relative poverty of rural households, including the head of household’s age, marital status, education level and the health status, as well as the number of household members aged 16 and below, the number of household members aged 65 and above, the proportion of males in the household, the maximum and minimum age of household members, temporary migration for work, and the household’s income-to-expenditure ratio. *α*_*m*_ is the regression coefficient of control variables. *ϕ*_*year*_ indicates the time fixed effects, *η*_*i*_ indicates the individual fixed effects, and *θ*_*province*_ indicates regional fixed effects.

### 4.2 Variable selection

We initially employ the probit model to evaluate the impact of rural household land transfer on the alleviation of relative poverty. However, since this model cannot account for individual fixed effects and may overlook the influence of time-invariant individual characteristics on the independent variables, we further employ both the two-way fixed effects model and the panel logit model to confirm the robustness of our findings.

Table 2 presents the variable descriptions of this paper. In variable selection, the dependent variable is whether the rural household is relatively poor in a given year. The core independent variable of our interest is engagement of rural households in land transfer. Given that the land transfer-in and transfer-out may have different effects on rural household relative poverty, this paper analyzes their impacts separately, which is shown in Table 2. In terms of control variables in Table 2, we include both head of household characteristics (age, marital status, education level, and health status) and household characteristics (number of household members aged 16 and below, number of household members aged 65 and above, the proportion of household males, maximum and minimum age of household members, temporary migration for work, and the income-to-expenditure ratio of household) to control for the effects of other variables on rural household relative poverty (Table 2). In addition, we consider inter-provincial factors by introducing the province dummy variable to control for inter-provincial level differences. We incorporate time fixed effects and individual fixed effects into the econometric model to control for time inherent characteristics that do not vary with individuals and individual inherent characteristics that do not change with time.

**Table 2:** Variable descriptions.

Categories	Variables	Variable descriptions
Dependent variable	Relative poverty	1 if reaches or exceeds MPI cutoff (0.33), 0 otherwise
Core independent variables	Land transfer	Whether the household engages in land transfer
	Land transfer-in	Whether the household engages in land transfer-in
	Land transfer-out	Whether the household engages in land transfer-out
Head of household characteristics	Age	Age of head of household
	Marital status	Married or living together is 1, otherwise is 0
	Education level	Number of years of education completed by the head of household
	Health status	1 if extremely healthy, 2 if very healthy, 3 if relatively healthy, 4 if average, 5 if unhealthy
Household characteristics	Number of household members aged 16 and under	Total number of household members aged 16 years and under in the household
	Number of household members aged 65 and above	Total number of household members aged 65 and over in the household
	Proportion of males in the household	Proportion of males in the household
	Maximum age of household members	Age of the oldest member of the household
	Youngest age of household members	Age of the youngest household member
	Temporary migration for work	1 if someone in the household temporarily migrate out of the area for work, otherwise it is 0
	Income-to-expenditure ratio of household	Total household expenditure for the year / total household income for the year

Table 3 presents descriptive statistics of relevant samples based on rural households’ engagement in the land transfer. We divide the samples into three categories: the full sample, the land transfer sub-sample, and the non-land transfer sub-sample. The table presents the means and standard deviations of the relevant variables, along with the differences in the means between the corresponding sub-samples. As shown in Table 3, the full sample has a relative poverty of 20.6%, emphasizing the importance of addressing relative poverty following China’s successful eradication of absolute poverty. Notably, the incidence of relative poverty in the land-transfer subsample is significantly lower than that in the non-land-transfer subsample with a 6.5% difference. There are significant differences between the two subsamples. Land-transferred households tend to have higher the maximum age of household members, a greater number of household members aged 16 and below, and older age of head of household. In addition, a higher percentage of heads of household are married and more household members are likely to temporarily migrate out of the area for work in land-transferred households than in non-land-transferred households. However, land-transferred households exhibit a lower income and expenditure ratio and poorer health status than that of non-land-transferred households. In conclusion, land transfer can significantly reduce the incidence of relative poverty.

**Table 3:** Summary statistics.

Variables	Full sample	Land transferred households	Non-land transferred households	Land transfer differences
Relative poverty	0.206	0.158	0.223	-0.065***
	(0.405)	(0.365)	(0.416)	(7.21)
Maximum age of household members	56.60	57.13	56. 42	0.709***
	(13.67)	(12.97)	(13.90)	(-3.74)
Youngest age of household members	23.87	23.80	23.89	-0.092
	(22.23)	(22.44)	(22.16)	(0.30)
Temporary migration for work	0.473	0.5	0.463	0.037***
	(0.499)	(0.5)	(0.499)	(-5.27)
Income-to-expenditure ratio of household	0.970	0.935	0.982	-0.047**
	(1.602)	(1.450)	(1.650)	(2.10)
Proportion of males in the household	0.515	0.513	0.515	-0.002
	(0.213)	(0.207)	(0.215)	(0.8)
Number of household members aged 16 and under	0.808	0.832	0.799	0.033**
	(0.980)	(0.988)	(0.977)	(-2.4)
Number of household members aged 65 and above	0.403	0.401	0.404	-0.003
	(0.678)	(0.675)	(0.679)	(0.31)
Age of head of household	47.63	48.41	47.36	1.045***
	(16.41)	(16.06)	(16.52)	(-4.55)
Marital status of head of household	0.819	0.826	0.816	0.010*
	(0.385)	(0.379)	(0.387)	(-1.79)
Education level of head of household	5.693	5.718	5.684	0.034
	(4.537)	(4.389)	(4.586)	(-0.57)
Health status of head of household	3.169	3.255	3.139	0.116***
	(1.261)	(1.247)	(1.265)	(-6.45)

Note

***, ** and * indicate significant at the 1%, 5%, and 10% levels, respectively; Standard errors of the estimated coefficients are in parentheses.

This paper conducted Pearson correlation tests on various variables, as shown in Table 4. Notably, there is a significant negative correlation between land transfer and relative poverty at the 1% level of significance. Maximum and youngest age of household members, income-to-expenditure ratio of household, number of household members aged 16 and under, number of household members aged 65 and above, age and health status of the head of household are significantly positively correlated with relative poverty; Temporary migration for work, proportion of males in the household, marital status and education level of the head of household are significantly negative correlated with relative poverty.

**Table 4:** Correlation tests results.

	Relative poverty	Land transfer	Maximum age of household members	Youngest age of household members	Temporary migration for work	Income-to-expenditure ratio of household	Proportion of males in the household	Number of household members aged 16 and under	Number of household members aged 65 and above	Age of head of household	Marital status of head of household	Education level of head of household	Health status of head of household
Relative poverty	1												
Land transfer	-0.070***	1											
Maximum age of household members	0.138***	0.023***	1										
Youngest age of household members	0.035***	-0.002	0.299***	1									
Temporary migration for work	-0.155***	0.032***	-0.061***	-0.230***	1								
Income-to-expenditure ratio of household	0.168***	-0.013**	0.040***	0.043***	-0.104***	1							
Proportion of males in the household	-0.041***	-0.00500	-0.035***	0.026***	0.020***	-0.021***	1						
Number of household members aged 16 and under	0.079***	0.014**	-0.081***	-0.657***	0.107***	-0.012*	-0.089***	1					
Number of household members aged 65 and above	0.135***	-0.002	0.698***	0.276***	-0.106***	0.039***	-0.018***	-0.079***	1				
Age of head of household	0.043***	0.028***	0.514***	0.509***	-0.111***	0.042***	0.003	-0.276***	0.381***	1			
Marital status of head of household	-0.080***	0.011*	0.008	-0.034***	0.039***	-0.017***	-0.032***	0.031***	0.022***	0.297***	1		
Education level of head of household	-0.175***	0.003	-0.185***	-0.112***	0.014**	-0.050***	0.037***	-0.030***	-0.138***	-0.161***	0.114***	1	
Health status of head of household	0.130***	0.040***	0.148***	0.147***	-0.028***	0.065***	-0.036***	-0.075***	0.093***	0.278***	0.014**	-0.174***	1

Note

***, ** and * indicate significant at the 1%, 5%, and 10% levels, respectively; Standard errors of the estimated coefficients are in parentheses.

To test whether there is multicollinearity among these variables, we conduct a variance inflation factor (VIF) test. The VIF values range from 1 to 2.47, which is significantly lower than 10, indicating that there is no multicollinearity problem among the variables.

## 5. Assessment of the poverty reduction effect of land transfer

### 5.1 Basic test

To investigate the effect of land transfer on the incidence of relative poverty among rural households, we conduct regressions using Eqs (1) and (2). These results are presented in Table 5, where the core independent variable, land transfer, is significant and stable. Column (1) shows the regression results of the two-way fixed effects model. Notably, the core independent variable is significantly negative at the 1% level of significance, indicating that relative poverty among rural households can be significantly reduced by conducting the land transfer. Given that the dependent variable, relative poverty, is a dummy variable, there are limitations when performing OLS regressions. To enhance the robustness of the results, we conduct the regressions using the probit model (column 2) and the panel logit model (column 3). Both columns show that the core independent variable is significantly negative, consistent with the regression results of the two-way fixed effects model. Therefore, land transfer effectively alleviates the relative poverty of rural households, demonstrating the robustness of our results.

**Table 5:** Effect of land transfer on the incidence of relative poverty among rural households.

	(1)	(2)	(3)
Land transfer	-0.042***	-0.059***	-0.395***
	(0.007)	(0.006)	(0.064)
Maximum age of household members	0.002***	0.002***	0.016***
	(0.001)	(0.001)	(0.003)
Youngest age of household members	-0.001*	0.001***	-0.004**
	(0.001)	(0.001)	(0.002)
Temporary migration for work	-0.090***	-0.104***	-0.727***
	(0.006)	(0.005)	(0.051)
Income-to-expenditure ratio of household	0.030***	0.205***	0.191***
	(0.002)	(0.001)	(0.015)
Proportion of males in the household	-0.076***	-0.033***	-0.641***
	(0.017)	(0.011)	(0.144)
Number of household members aged 16 and under	0.016***	0.043***	0.121***
	(0.006)	(0.003)	(0.046)
Number of household members aged 65 and above	0.033***	0.036***	0.266***
	(0.007)	(0.005)	(0.059)
Age of head of household	-0.001	-0.001***	-0.005*
	(0.001)	(0.001)	(0.003)
Marital status of head of household	-0.016*	-0.047***	-0.111
	(0.010)	(0.007)	(0.081)
Education level of head of household	-0.001	-0.011***	-0.008
	(0.001)	(0.001)	(0.008)
Health status of head of household	0.015***	0.030***	0.124***
	(0.002)	(0.002)	(0.021)
Individual fixed effects	YES	NO	YES
Time fixed effects	YES	YES	YES
Regional fixed effects	NO	YES	NO
Constant	0.191***	-0.528	
	(0.026)	(0.336)	
Observations	25590	25590	11267
R^2^ or Adjusted R^2^	0.294	0.125	0.11

Note

***, ** and * indicate significant at the 1%, 5%, and 10% levels, respectively; Standard errors of the estimated coefficients are in parentheses.

Next, we analyze the effect of each control variable on the incidence of relative poverty based on the regression results of the probit model. As shown in column (2), among the household characteristics, the maximum and the minimum age of household members, the household’s income-to-expenditure ratio, the number of people aged 16 and below, and the number of people aged 65 and above all demonstrates significant positive effects. These results indicate that household with the older maximum age of household members, younger minimum age of household members, higher income-to-expenditure ratio, and larger the number of household members aged 16 and below and 65 and above are more likely to experience relative poverty. This may be attributed to the increased responsibility of such households in providing childcare and elder assistance. Moreover, a higher household income-to-expenditure ratio suggests a smaller proportion of annual savings, making the household less resilient to unexpected financial challenges.

Conversely, variables related to household members temporarily migrating for work and the proportion of males in the household exhibit significant negative effects, indicating that households with members temporarily working outside the area and a higher proportion of males are less likely to experience relative poverty. This could be attributed to the higher likelihood of male household members contributing to increased income through temporary work migration, thus improving household conditions and reducing the incidence of relative poverty.

The health status of the head of the household is a significant factor with a positive impact on relative poverty. This finding suggests that in rural households, the likelihood of experiencing relative poverty increases when the head of the household’s health is poor. Typically, the head of household is the primary labor and income source for the family. When the health of the head of household is poor, it places a higher financial burden on the household. The marital status, age, and education level of head of household have significant negative effects. As married households are likely to have a more complete and stable family structure, they are less likely to face relative poverty. Younger head of household are often in their prime working years, contributing to a higher household income. Additionally, a higher level of education of head of the household is associated with increased income, further mitigating the risk of relative poverty.

### 5.2 Robustness tests

#### 5.2.1. Robustness test—replacement of independent variables

Relative poverty depth reflects the gap between the relatively poor population and the poverty cutoff, making it a valuable indicator for determining relative poverty status to a significant extent. In this study, we substitute relative poverty depth for the dependent variable and conduct regressions using Eqs (1) and (2). As shown in Table 6, the core independent variable, land transfer, is significantly negative at the 1% level of significance, affirming that land transfer significantly reduces relative poverty depth, aligning with the key findings from basic test.

**Table 6:** Robustness test—replace independent variables as relative poverty depth.

	Two-way fixed effects model	Probit model (marginal effect)
Independent variables	Land transfer	Land transfer
Coefficients	-0.016***	-0.002***
Standard errors	0.002	0.002
Control variables	YES	YES
Time fixed effects	YES	YES
Regional fixed effects	NO	YES
Individual fixed effects	YES	NO
Observations	25590	25590
R^2^ or Adjusted R^2^	0.25	0.18

Note

***, ** and * indicate significant at the 1%, 5%, and 10% levels, respectively; Standard errors of the estimated coefficients are in parentheses.

#### 5.2.2. Robustness test-using instrumental variables to overcome endogeneity

Considering that there is a certain degree of self-selection in whether rural households engage in land transfer, this paper selects the land transfer rate of other households in the same village as an instrumental variable to address the endogeneity problem. We select this instrumental variable because it satisfies the exogeneity requirement by correlating with whether the household transfer land, while having no direct impact on the household’s relative poverty status.

As shown in Table 7, the land transfer rate of other households in the same village is significantly positive at the 1% level of significance. It implies that a household is more likely to transfer its land if other households in the same village do so at a higher rate. Therefore, there is strong correlation between these two variables. The Hansen J statistic of the model is zero, so it is not over-identified. In addition, the F-statistic used to test the weak instrumental variable is 2028.953, allowing us to reject the null hypothesis that the instrumental variable is weak.

**Table 7:** Robustness tests—instrumental variable method.

	(1)	(2)	(3)	(4)
Land transfer		-0.153*** (0.018)		-0.722*** (0.077)
Land transfer rate of other households in the same village	0.713*** (0.016)		0.679*** (0.013)	
Control variables	YES	YES	YES	YES
Time fixed effects	YES	YES	YES	YES
Regional fixed effects	YES	YES	YES	YES
Constant	-0.022 (0.022)	0.213*** (0.021)	-0.141 (0.105)	-0.589* (0.341)
Observations	25590	25590	25590	25590

Note

***, ** and * indicate significant at the 1%, 5%, and 10% levels, respectively; Standard errors of the estimated coefficients are in parentheses.

Columns (1) and (2) of Table 7 show the regression results of the first and second stages of the IV regression model. The coefficient of land transfer in column (2) is significantly negative at the 1% level of significance, indicating that land transfer effectively reduces the incidence of relative poverty among rural households. Similarly, columns (3) and (4) show the regression results of the first and second stages of the IV probit model, with column (4) revealing a significantly negative effect of land transfer at the 1% significance level. It further suggests that after addressing the self-selective endogeneity of land transfer by rural households, land transfer continues to be effective in reducing the incidence of relative poverty among rural households.

#### 5.2.3. Robustness test—adjusting the cutoff value *K*

When identifying whether rural households experience relative poverty, we apply an MPI cutoff *K* = 0.33. To verify that the poverty reduction effect of land transfer is universal under different cutoffs, we adjust the cutoff value multiple times to test the robustness of our previous results. Fig 1 shows that the incidence of relative poverty is extremely high when the cutoff *K*<0.2, especially at the cutoff *K* = 0.1, reaching nearly 90%; when the cutoff *K*>0.5, the relative poverty incidence is extremely low and approaching zero. In light of the above circumstances, we examine the impact of land transfer on the incidence of relative poverty in rural households by setting the cutoff K at various values, such as 0.2, 0.3, 0.4, and 0.5. The results are presented in Table 8.

**Table 8:** Robustness tests—adjusting the cutoff K.

	Two-way fixed effects model				Probit model			
	*K* = 0.2	*K* = 0.3	*K* = 0.4	*K* = 0.5	*K* = 0.2	*K* = 0.3	*K* = 0.4	*K* = 0.5
Land transfer	-0.050***	-0.054***	-0.030***	-0.009***	-0.206***	-0.254***	-0.275***	-0.326***
	(0.009)	(0.008)	(0.005)	(0.003)	(0.019)	(0.021)	(0.030)	(0.052)
Control variables	YES	YES	YES	YES	YES	YES	YES	YES
Time fixed effects	YES	YES	YES	YES	YES	YES	YES	YES
Regional fixed effects	NO	NO	NO	NO	YES	YES	YES	YES
Individual fixed effects	YES	YES	YES	YES	NO	NO	NO	NO
Constant	0.525***	0.293***	0.112***	0.025**	0.602	0.021	-0.438	-1.078**
	(0.031)	(0.028)	(0.019)	(0.010)	(0.370)	(0.324)	(0.349)	(0.453)
Observations	25590	25590	25590	25590	25590	25590	25590	25488
R^2^ or Adjusted R^2^	0.16	0.17	0.11	0.05	0.11	0.12	0.12	0.14

Note

***, ** and * indicate significant at the 1%, 5%, and 10% levels, respectively; Standard errors of the estimated coefficients are in parentheses.

Table 8 shows that the regression coefficient of land transfer is significantly negative at the 1% significance level in both the two-way fixed effects model and the probit model. This consistent result reinforces the conclusion that land transfer significantly reduces the incidence of relative poverty among rural households. Hence, adjusting the cutoff value K does not alter the robustness of our previous conclusion.

#### 5.2.4. Robustness test—changing the weight assignment method

To further verify the robustness of the findings under different weighting methods, we adopt the equal weight method for all indicators. When we recalculate the MPI and regress Eqs (1) and (2) to investigate the effect of the land transfer on the relative poverty of rural households, the results in Table 9 confirm that the regression coefficients of land transfer are significantly negative. In the two-way fixed effects model, they are at the 5% level of significance, and in the probit model, they are at the 1% level of significance. These results reinforce that land transfer effectively reduce the incidence of relative poverty among rural households, supporting our earlier findings.

**Table 9:** Robustness test—equal weight method.

	(1) Two-way fixed effects model	(2) Probit model
Land transfer	-0.022**	-0.144***
	(0.009)	(0.037)
Control variables	YES	YES
Time fixed effects	YES	YES
Regional fixed effects	NO	YES
Individual fixed effects	YES	NO
Constant	0.058	-0.973***
	(0.043)	(0.147)
Observations	25590	25590
R^2^ or Adjusted R^2^	0.15	0.16

Note

***, ** and * indicate significant at the 1%, 5%, and 10% levels, respectively; Standard errors of the estimated coefficients are in parentheses.

#### 5.2.5. Robustness test—excluding dimensions

When constructing the MPI in this paper, we improve the MPI proposed by the UNDP by extending three dimensions (health, education, and living status) to eight dimensions. To ensure the robustness of our findings and confirm that they are not solely attributed to any specific dimension, we exclude one dimension at a time and recalculated the MPI. The results of the regression analysis using Eqs (1) and (2) are shown in Tables 10 and 11.

**Table 10:** Robustness test—removing dimensions (two-way fixed effects model).

	Remove employment	Remove health care	Remove housing	Remove land	Remove income
Land transfer	-0.046***	-0.046***	-0.050***	-0.019**	-0.050***
	(0.007)	(0.007)	(0.008)	(0.007)	(0.007)
Control variables	YES	YES	YES	YES	YES
Time fixed effects	YES	YES	YES	YES	YES
Regional fixed effects	NO	NO	NO	NO	NO
Individual fixed effects	YES	YES	YES	YES	YES
Constant	0.180***	0.209***	0.212***	0.151***	0.288***
	(0.025)	(0.026)	(0.029)	(0.027)	(0.027)
Observations	25590	25590	25590	25590	25590
R^2^	0.16	0.21	0.17	0.16	0.07

Note

***, ** and * indicate significant at the 1%, 5%, and 10% levels, respectively; Standard errors of the estimated coefficients are in parentheses.

**Table 11:** Robustness test—removing dimensions (Probit model).

	Remove employment	Remove health care	Remove housing	Remove land	Remove income
Land transfer	-0.271***	-0.280***	-0.244***	-0.111***	-0.268***
	(0.024)	(0.043)	(0.045)	(0.032)	(0.042)
Control variables	YES	YES	YES	YES	YES
Time fixed effects	YES	YES	YES	YES	YES
Regional fixed effects	YES	YES	YES	YES	YES
Individual fixed effects	NO	NO	NO	NO	NO
Constant	-0.652*	-0.333***	-0.497***	-0.351***	0.488***
	(0.341)	(0.128)	(0.097)	(0.101)	(0.139)
Observations	25590	25590	25590	25590	25590
Adjusted R^2^	0.12	0.16	0.13	0.15	0.07

Note

***, ** and * indicate significant at the 1%, 5%, and 10% levels, respectively; Standard errors of the estimated coefficients are in parentheses.

Table 10 shows the regression results under the two-way fixed effects model by excluding the employment, health care, housing, land, and income dimensions. When excluding the land dimension, the land transfer is significantly negative at the 5% level of significance. Moreover, when we exclude the remaining five dimensions separately, the land transfer remains significantly negative at the 1% level of significance. In Table 11 the results from the probit model consistently demonstrate that land transfer is significantly negative at the 1% level of significance. The above results indicate that land transfer substantially reduces the incidence of relative poverty among rural households, further validating the robustness of the previous results.

### 5.3 Heterogeneity analysis

We divide the data into two subsets of land transfer-in and land transfer-out in light of the possibility that different land transfer methods may affect the relative poverty incidence of rural households. Then, we regress Eqs (1) and (2) using a panel logit model, a probit model, and a two-way fixed effects model, the results are shown in Table 12. In Table 12, columns (1) through (3) show the effect of land transfer-in on the relative poverty incidence of rural families, and columns (4) through (6) show the effect of land transfer-out on this same indicator. According to the findings, land transfer-in and transfer-out are significantly negative at the 1% level of significance, demonstrating that both methods can substantially lower the relative poverty incidence of rural families. Column (2) shows that land transfer-in reduces the likelihood of relative poverty in rural households by 4%, whereas column (5) shows that land transfer-out reduces the likelihood of relative poverty in rural households by 7.1%. As a result, the land transfer-out has a more significant impact on reducing poverty than transfer-in (Table 12).

**Table 12:** Heterogeneity analysis of the effect of land transfer on relative poverty.

	(1) Two-way fixed effects model	(2) Probit model (marginal effect)	(3) Panel logit model	(4) Two-way fixed effects model	(5) Probit model (marginal effect)	(6) Panel logit model
Land transfer-in	-0.029***	-0.040***	-0.259***			
	(0.009)	(0.007)	(0.087)			
Land transfer-out				-0.052***	-0.071***	-0.510***
				(0.009)	(0.007)	(0.085)
Control variables	YES	YES	YES	YES	YES	YES
Individual fixed effects	YES	NO	YES	YES	NO	YES
Time fixed effects	YES	YES	YES	YES	YES	YES
Regional fixed effects	NO	YES	NO	NO	YES	NO
Constant	0.189***	-0.517		0.191***	-0.514	
	(0.026)	(0.335)		(0.026)	(0.336)	
Observations	25590	25590	11267	25590	25590	11267
R^2^ or Adjusted R^2^	0.15	0.12	0.11	0.29	0.13	0.10

Note

***, ** and * indicate significant at the 1%, 5%, and 10% levels, respectively; Standard errors of the estimated coefficients are in parentheses.

The two plausible hypotheses offered in this research are: (i) land transfer-out and non-agricultural employment have a positive relationship, while land transfer-in and non-agricultural employment have a negative relationship. Land transfer-out can dramatically lower the relative poverty by allowing rural households to work in cities and access higher income opportunities, better educational and medical resources. (ii) Land transfer-out reduces the relative poverty of rural households more effectively than land transfer-in since households, as land transfer-in experience more production cost limitations and other uncertainties.

## 6. Further analysis

### 6.1 Setting of the econometric model

This paper constructs the following two-way fixed effects model based on the least squares method to explore the long-term effect of land transfer on relative poverty among rural households.

$$Poor_{it}=\alpha_{0}+\beta transfer_{it-j}+\sum_{n}\alpha_{m}{X^{m}}_{it}+\phi_{year}+\eta_{i}+\delta_{it},\;i=1,2,\ldots ,N$$ (4)

*j* represents the lag order of the variable *transfer*_*it*_,and the meanings of other variables are consistent with the previous paper.

### 6.2 Analysis of the mechanism of land transfer to alleviate relative poverty

The previous section has established that land transfer can lower the relative poverty incidence of rural households, with land transfer-out has a greater effect on reducing poverty than land transfer-in. However, further research is required to determine which specific dimensions of land transfer lowers the overall relative poverty incidence. This paper uses the incidence of relative poverty in each dimension as the dependent variable, and examines the effects of land transfer on education, health, employment, health care, quality of life, housing, land, and income dimensions using a two-way fixed effects model. It then investigates the methods of land transfer to reduce the relative poverty incidence of rural households. Considering that the methods of land transfer have different effects on the efficiency and welfare of agricultural land resource allocation, this paper examines the mechanisms of poverty reduction for land transfer-in and land transfer-out separately, and the results are shown in Tables 13–16.

**Table 13:** Analysis of land transfer-in mechanisms for poverty reduction (1).

	(1)	(2)	(3)	(4)
	Education	Health	Employment	Health care
Land transfer-in	0.015**	0.003	0.002	-0.003
	(0.004)	(0.005)	(0.008)	(0.006)
Control variables	YES	YES	YES	YES
Time fixed effects	YES	YES	YES	YES
Individual fixed effects	YES	YES	YES	YES
Constant	0.139***	-0.072***	0.266***	0.120***
	(0.022)	(0.015)	(0.017)	(0.018)
Observations	25590	25590	25590	25590
R^2^	0.19	0.18	0.03	0.03

Note

***, ** and * indicate significant at the 1%, 5%, and 10% levels, respectively; Standard errors of the estimated coefficients are in parentheses.

**Table 14:** Analysis of land transfer-in mechanisms for poverty reduction (2).

	(5)	(6)	(7)	(8)
	Quality-of-life	Housing	Land	Income
Land transfer-in	0.006	-0.007***	-0.016***	-0.001
	(0.004)	(0.001)	(0.003)	(0.003)
Control variables	YES	YES	YES	YES
Time fixed effects	YES	YES	YES	YES
Individual fixed effects	YES	YES	YES	YES
Constant	0.119***	0.179***	0.277***	0.034***
	(0.011)	(0.007)	(0.021)	(0.006)
Observations	25590	25590	25590	25590
R^2^	0.02	0.04	0.05	0.2

Note

***, ** and * indicate significant at the 1%, 5%, and 10% levels, respectively; Standard errors of the estimated coefficients are in parentheses.

**Table 15:** Analysis of land transfer-out mechanisms for poverty reduction (1).

	(1)	(2)	(3)	(4)
	Education	Health	Employment	Health care
Land transfer-out	0.009	0.009	0.009	0.001
	(0.008)	(0.006)	(0.007)	(0.004)
Control variables	YES	YES	YES	YES
Time fixed effects	YES	YES	YES	YES
Individual fixed effects	YES	YES	YES	YES
Constant	0.139***	-0.072***	0.266***	0.120***
	(0.022)	(0.015)	(0.017)	(0.018)
Observations	25590	25590	25590	25590
R^2^	0.18	0.16	0.03	0.02

Note

***, ** and * indicate significant at the 1%, 5%, and 10% levels, respectively; Standard errors of the estimated coefficients are in parentheses.

**Table 16:** Analysis of land transfer-out mechanisms for poverty reduction (2).

	(5)	(6)	(7)
	Quality-of-life	Housing	Income
Land transfer-out	-0.016***	-0.003	-0.004
	(0.003)	(0.003)	(0.003)
Control variables	YES	YES	YES
Time fixed effects	YES	YES	YES
Individual fixed effects	YES	YES	YES
Constant	0.119***	0.179***	0.034***
	(0.011)	(0.007)	(0.006)
Observations	25590	25590	25590
R^2^	0.03	0.03	0.19

Note

***, ** and * indicate significant at the 1%, 5%, and 10% levels, respectively; Standard errors of the estimated coefficients are in parentheses.

The results in Tables 13 and 14 reveal that land transfer-in is insignificant at the 10% significance level in the health, employment, health care, quality-of-life, and income dimensions, indicating that land transfer-in does not significantly lower the incidence of poverty in these dimensions. This suggests that land transfer-in does not considerably improve rural household members’ health, employment, medical insurance participation, income, and quality-of-life. Furthermore, land transfer-in increases the incidence of poverty in the education dimension as the coefficient is significant positive at the 5% significance level. It might be that land transfer-in can increase the labor intensity of a rural household, leading to insufficient adults to work and forcing children to drop out of school to work at home (Table 13). Lastly, land transfer-in is significantly negative in the housing and land dimensions at the 1% significance level, which indicates that it effectively reduces poverty in those dimensions. This may be because land transfer-in increase the land area of rural households and thus increase the housing area per capita and improve land condition (Table 14).

This paper will not examine the impact of land transfer-out on poverty reduction in the land dimension since households that engage in land transfer-out lose their contractual rights to land. Firstly, Tables 15 and 16 demonstrate that land transfer-out is insignificant in the education, health, employment, health care, housing, and income dimensions at the 10% significance level (Tables 15 and 16). This finding implies that land transfer-out does not significantly lower the incidence of poverty in these dimensions. It also suggests that land transfer-out does not substantially improve rural households’ education, health, employment, medical insurance participation, housing, and income. Secondly, the land transfer-out is significantly negative in the quality-of-life dimension at the 1% significance level, showing that it substantially improves the quality-of-life of rural households and thereby significantly lower the incidence of poverty. It is possible that households that engage in land transfer-out move to urban areas, where typically offer better living conditions than rural areas and can reduce relative poverty.

### 6.3 The long-term impact of land transfer on relative poverty

To explore the long-term effect of land transfer on relative poverty among rural households, we introduce first-order and second-order lag terms as independent variables. As the panel data is in collected biennially, the first-order and second-order lag terms represent the effect of land transfer on relative poverty among rural households after two and four years. Column (1) of Table 17 shows the regression results of relative poverty on the first-order lag term of land transfer. The core independent variable is significantly negative at the 10% level of significance, indicating that after two years of land transfer, the relative poverty among rural households continues to significantly decrease. Column (2) of Table 17 shows the regression results of relative poverty on the second -order lag term of land transfer. The core independent variable lacks statistical significance, indicating that after four years of land transfer, the relative poverty among rural households cannot be significantly reduced. This may be because the reduction in the incidence of relative poverty among rural households due to land transfer may have already reached its maximum effect during the second to fourth year.

**Table 17:** The long-term impact of land transfer on relative poverty.

	(1) Two-way fixed effects model	(2) Two-way fixed effects model
L. Land transfer	-0.011* (0.006)	
L2. Land transfer		-0.018 (0.013)
Control variables	YES	YES
Time fixed effects	YES	YES
Individual fixed effects	YES	YES
Constant	0.173***	0.119***
	(0.019)	(0.039)
Observations	20078	13306
R^2^	0.15	0.14

Note

***, ** and * indicate significant at the 1%, 5%, and 10% levels, respectively; Standard errors of the estimated coefficients are in parentheses.

## 7. Conclusion and recommendation

This study uses data from the CFPS database from 2012 to 2018 to identify and assess the relative poverty of rural families in China by creating a MPI from a multidimensional perspective. The findings indicate that China’s poverty alleviation measures have made a substantial impact with the annual reduction in relative poverty in rural areas observed from 2012 to 2018. However, relative poverty remains a vital issue in China’s rural areas, as seen by the overall incidence of rural households in relative poverty, which is 20.6%. These results align with the research of Deng and Zheng (2022) [15]. In the past, income-related concerns were the main focus of China’s efforts to combat poverty. The focus of China’s poverty alleviation efforts should be shifted from emphasizing on income to a multidimensional approach including education, health, and employment prior to the current relative poverty problem in rural areas. This will contribute to reducing the relative poverty of rural households.

Additionally, land is an important asset for rural families and has a significant impact on their economic status. Land transfer serves as a crucial measure of rural land reform, fostering agricultural development and consequently increasing the income of rural households. As a result, the relative poverty of rural families could decrease. This research provides an empirical analysis of the impact of land transfer on reducing poverty. The findings demonstrate that land transfer can greatly lower the incidence of relative poverty among rural families, which is supported by multiple robustness tests. Hence, China should actively promote land transfer among rural households through appropriate policies. Compared with Liu and Wang (2020), Zhou et al (2020), Zuo and Lu (2020) [18–20], this paper takes a more comprehensive approach to measure poverty by including dimensions of education, health, employment, health care, quality of life, housing, and land, in addition to income. Unlike Li et al (2021) [21], which did not address endogeneity, potentially leading to biased results, our paper overcomes the endogeneity between land transfer and relative poverty among households, contributing to more scientific results.

As two methods of land transfer, both land transfer-in and land transfer-out effectively reduce the relative poverty of rural households. Land transfer-out has a greater impact on reducing poverty than land transfer-in, which is consistent with the research results of Zhou et al (2020) [19]. Therefore, more favorable policies should be offered to households that are acquiring land, lowering the cost of maintaining the land and helping them in expanding the land to increase profits. In addition, while Liu and Wang (2020) also examined the poverty reduction mechanism of land transfer [18], they did not explore the differences in poverty reduction mechanisms between land transfer-in and land transfer-out. This paper reveals that land transfer-in and land transfer-out have different mechanisms to reduce poverty. Land transfer-in reduces the relative poverty incidence of rural households mainly through the housing dimension and the land dimension, while land transfer-out primarily affects the quality-of-life dimension. Furthermore, our research is the first to reveal that the reduction effect of land transfer on the incidence of relative poverty among rural households persists for at least two years, but by the fourth year, this effect disappears.

The land reform system currently implemented in China optimizes the structure of the agricultural labor force, liberates rural productivity, and enhances the ability of rural households to resist poverty in many ways. Therefore, China should further consolidate and improve the land reform system to enhance the long-term effectiveness and sustainability of land transfer to alleviate relative poverty.
